# Gastroprotective mechanism of modified lvdou gancao decoction on ethanol-induced gastric lesions in mice: Involvement of Nrf-2/HO-1/NF-κB signaling pathway

**DOI:** 10.3389/fphar.2022.953885

**Published:** 2022-09-01

**Authors:** Lei Xie, Minyi Luo, Junlin Li, Wenguan Huang, Guangjun Tian, Xiuyun Chen, Ying Ai, Yan Zhang, Haolan He, Jinyang He

**Affiliations:** ^1^ Science and Technology Innovation Center, Guangzhou University of Chinese Medicine, Guangzhou, Guangdong, China; ^2^ Liver Diseases Center, Guangdong Provincial Hospital of Chinese Medicine, Zhuhai, Guangdong, China; ^3^ Artemisinin Research Center, Guangzhou University of Chinese Medicine, Guangzhou, Guangdong, China; ^4^ First Clinical Medical College, Guangzhou University of Chinese Medicine, Guangzhou, Guangdong, China; ^5^ Guangzhou Eighth People’s Hospital, Guangzhou, Guangdong, China

**Keywords:** ethanol-induced gastric lesions, lvdou gancao decoction, ROS, vasodilation, inflammatory response, oxidative stress

## Abstract

Modified Lvdou Gancao decoction (MLG), a traditional Chinese medicine formula, has been put into clinical use to treat the diseases of the digestive system for a long run, showing great faculty in gastric protection and anti-inflammatory, whereas its protective mechanisms have not been determined. The current study puts the focus on the protective effect and its possible mechanisms of MLG on ethanol-induced gastric lesions in mice. In addition to various gastric lesion parameters and histopathology analysis, the activities of a list of relevant indicators in gastric mucosa were explored including ALDH, ADH, MDA, T-SOD, GSH-Px, and MPO, and the mechanisms were clarified using RT-qPCR, ELISA Western Blot and immunofluorescence staining. The results showed that MLG treatment induced significant increment of ADH, ALDH, T-SOD, GSH-Px, NO, PGE2 and SS activities in gastric tissues, while MPO, MDA, TNF-α and IL-1β levels were on the decline, both in a dose-dependent manner. In contrast to the model group, the mRNA expression of Nrf-2 and HO-1 in the MLG treated groups showed an upward trend while the NF-κB, TNFα, IL-1β and COX2 in the MLG treated groups had a downward trend simultaneously. Furthermore, the protein levels of p65, p-p65, IκBα, p-IκBα, iNOS, COX2 and p38 were inhibited, while Nrf2, HO-1, SOD1, SOD2 and eNOS were ramped up in MLG treatment groups. Immunofluorescence intensities of Nrf2 and HO-1 in the MLG treated groups were considerably enhanced, with p65 and IκBα diminished simultaneously, exhibiting similar trends to that of qPCR and western blot. To sum up, MLG could significantly ameliorate ethanol-induced gastric mucosal lesions in mice, which might be put down to the activation of alcohol metabolizing enzymes, attenuation of the oxidative damage and inflammatory response to maintain the gastric mucosa. The gastroprotective effect of MLG might be achieved through the diminution of damage factors and the enhancement of defensive factors involving NF-κB/Nrf2/HO-1 signaling pathway. We further confirmed that MLG has strong potential in preventing and treating ethanol-induced gastric lesions.

## Graphical Abstract

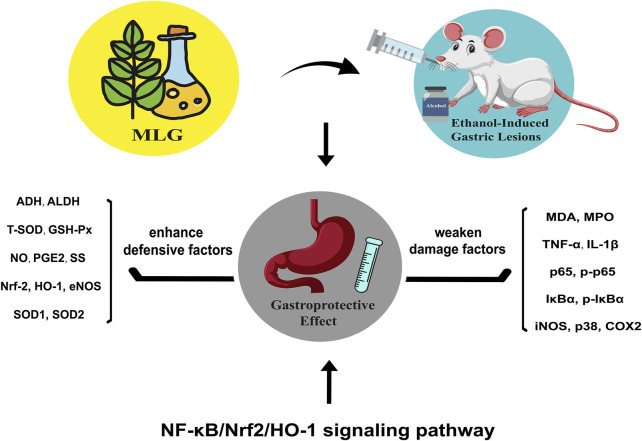

## Introduction

Excessive alcohol consumption can pose serious challenges to a cascade of vital organs, including the liver, brain, heart, and gastrointestinal tract (Hyun et al., 2021). Similar to chronic abuse, acute alcohol consumption is toxic to many organs, the stomach being particularly vulnerable in this process, predisposing to gastrointestinal disorders, including acute gastric mucosal lesion (AGML), gastritis, gastric ulcers, and gastric bleeding (Gonçalves et al., 2017). The role of gastroprotective effect in protecting the body against alcohol-related toxicity, oxidative stress as well as inflammation response cannot be overlooked.

Ethanol could result in severe gastric mucosal damage through direct toxic action or free radicals to activate oxidative stress, leading to acute hemorrhagic lesions, edema of the mucus membrane, epithelial exfoliation and the release of inflammatory cells (Wang et al., 2015; Li J et al., 2017). This acute gastric mucosal damage may be attributed to the low activity of defensive factors such as nitric oxide (NO), prostaglandins (PG), mucosal microcirculation, antioxidants and certain cytokines, or is due to the overproduction of reactive oxygen species (ROS), lipid peroxidation and infiltration of neutrophils (Escobedo-Hinojosa et al., 2018; Magierowska et al., 2018; Zaghlool et al., 2019). NO, working as a mixed blessing in the alimentary system, is synthesized with the participation of nitric oxide synthase (NOS) (Mohamed et al., 2022). It is widely recognized that, in the digestive system, NO generated by constitutive NOS (cNOS) is related to cytoprotective effect, whereas inducible NOS (iNOS)-produced NO, by contrast, is cytotoxic (Motawi et al., 2007). Excessive alcohol consumption may intensify gastric mucosal damage via down-modulating the levels of defensive factors, represented by NO and prostaglandin E2 (PGE2), which contribute to the improvement of gastric mucosal blood flow and mucosal microcirculation under normal conditions (Xie et al., 2019).

Several studies reported that oxidative stress and inflammatory responses play a significant role in the development and progression of alcohol-induced gastric mucosal damage(Arda-Pirincci et al., 2006; Pan et al., 2008; Wu et al., 2018). As two interplaying systems regulating the equilibrium of cellular redox status, Nrf-2/HO-1 signaling axis and NF-κB could modulate both oxidative stress and inflammatory response indeed (Casili et al., 2020; Kim et al., 2021). They express at relatively low levels normally while are upregulated under stress conditions. It is well-founded that Nrf2/HO-1 signaling are protecting factors against oxidative stress and inflammatory responses in gastric mucosal damage, while NF-κB plays an opposite role (Dimauro et al., 2021). Furthermore, Nrf2 activation and its anti-inflammatory effect are closely related to the transcription of antioxidant factor through NF-κB. Deficiency of Nrf2 could increase the activity of NF-κB, leading to increased cytokine production associated with increased oxidative stress (Sandberg et al., 2014). The production and removal of ROS equilibrates dynamically in the gastric mucosa under the physiology condition (Lebda et al., 2018). Induced by a series of factors such as ethanol exposure, inflammatory stimuli lead to the excessive production of ROS along with the decline of antioxidative enzymes, which disrupts the equilibrium of oxidation and anti-oxidation systems, thereby supervening the gastric lesions (Jeon et al., 2014). Considering the molecular crosstalk of NF-κB/Nrf-2/HO-1 is indispensable for the ROS-mediated inflammatory cascades and the regulation of ethanol-induced gastric lesions, it is fair enough to believe that mitigating the NF-κB activation or supporting Nrf2 activation may be effective strategies for treatment of ethanol-induced gastric lesions. It is well established that ethanol and metabolite acetaldehyde would attack the gastric mucosa, inducing microcirculatory disturbance and hypoxia, along with consequent propagation of the inflammatory cascade (De Araújo et al., 2018; Yu Y et al., 2020). ADH and ALDH, as crucial alcohol metabolism enzymes, could oxidize acetaldehyde to harmless acetic acid to accelerate alcohol metabolism and ameliorate the ethanol-induced gastric lesions. Some natural plant extracts have been shown to activate ADH and ALDH in previous studies (Martin and Maricle, 2015; Jang et al., 2018).

Current common-used treatments for ethanol-induced gastric lesions treatment largely include potentiating the protective mechanism of gastric mucosa and improving the gastric mucosal microcirculation to repair of gastric mucosa (Association, 2015). H2 receptor antagonists, such as cimetidine, roxatidine, ranitidine, and famotidine, continue to be the first-line therapy for peptic ulcer disease, which has been proven effective for AGML through suppressing gastric acid secretion. However, in ethanol-induced AGML, some H2 receptor antagonists could block gastric first-pass ethanol metabolism, resulting in slightly higher blood alcohol levels than normal after consuming alcohol (Weathermon and Crabb, 1999; Breslow et al., 2015). Colloid Bismuth Pectin (CBP) could protect the gastric mucosa by reacting with the complexes of biOCl and bismuth citrate to form a protective film (Li and Sun, 2012; Adeyemi and Onwudiwe, 2020). Nevertheless, the side effect of CBP is not to be ignored that grumbles from the users about constipation and other adverse reactions have gained prominence. Therefore, it is of great significance to screen out better drugs, particularly extracted from plants or herbs which display higher efficiency and lower toxicity.

The research on natural products is all the rage for their great efficacy and low toxicity, and the natural compounds they contain have been regarded as superior compatibility with the human body (Ashktorab et al., 2019). The first appearance of Lvdou Gancao decoction (LGD), a famous traditional Chinese medicine prescription, was found in Wen Cheng’s *Jijiu Bianfang* in the Qing Dynasty. LGD exerts a good detoxification effect in traditional use and has a reliable therapeutic effect against acute organophosphorus pesticide poisoning (AOPP) (Cailin, 1999; Wang, 2002), drug poisoning (Zhou, 2009; Wenxue, 2018), mushroom poisonings (Zhang, 2000) and digestive system diseases such as toxic hepatitis (Li and Tang, 1997; Wenquan, 2007; Weifeng and Jia, 2022) and acute pancreatitis (Wei et al., 2013). Based on this classic LGD, the modified Lvdou Gancao decoction (MLG) is made of several components and could be effective in gastropathy (Minqing and Mingwei, 2002; Dan et al., 2018; Yuewen and Qiang, 2018), liver complaints (Wang, 2017; Xie et al., 2022) and alcohol-induced conditions (Sun, 2015; Takei et al., 2015; Zhang and Zhang, 2017). Many of the herbs applied by MLG have been put into clinical use to treat the diseases of the digestive system for a long run and have shown their faculty in gastric protection and anti-inflammatory (Wang et al., 2012; Lv et al., 2018; Cao et al., 2020; Meng et al., 2020). Although their therapeutic mechanisms have not yet been known, their therapeutic efficacy has been speculated to be largely related to their antioxidant effect, for example, Dangshen(Wang et al., 1997; Li L et al., 2017), Shanyao (Qiao et al., 2018), Dingxiang (Agboola et al., 2022), Banxia (Yu L et al., 2020; Fu et al., 2021), Tianma(Dong et al., 2021), and Shengjiang (Haniadka et al., 2013). Previous studies indicated these herbs can effectively remove free radicals and inhibit the occurrence of lipid peroxidation, so as to protecting gastrointestinal mucosa. Our previous study has confirmed that MLG can efficaciously ameliorate alcohol-induced hepatotoxicity, accelerate the metabolism of alcohol and weaken inflammatory and oxidative stress responses in mice liver (Xie et al., 2022). The current study shifted its focus towards demonstrating the gastroprotective effect and mechanisms of MLG to further explore the value of clinical applications of MLG.

## Materials and methods

### Preparation and composition of MLG

Modified Lvdou Gancao decoction (MLG) is similar to the one in our previous studies (Xie et al., 2022), all herbs come from the same preparation and share the same batch number respectively. MLG consists of a mixture of 14 well-defined herbs, namely, Lvdou (Vigna radiata (L.) R.Wilczek [Fabaceae]); Gancao (Glycyrrhiza glabra L. [Fabaceae]); Baishao (Paeonia lactiflora Pall. [Paeoniaceae]); Huang Jiezi (Brassica juncea (L.) Czern. [Brassicaceae]); Chuanxiong (Conioselinum anthriscoides 'Chuanxiong' [Apiaceae]); Gansong (Nardostachys jatamansi (D.Don) DC. [Caprifoliaceae]); Dangshen (Codonopsis pilosula (Franch.) Nannf. [Campanulaceae]); Lianfang (Nelumbo nucifera Gaertn. [Nelumbonaceae]); Shanyao (Dioscorea oppositifolia L. [Dioscoreaceae]); Dingxiang (Syzygium aromaticum (L.) Merr. & L.M.Perry [Myrtaceae]); Jiangbanxia (Pinellia ternata (Thunb.) Makino [Araceae]); Tianma (Gastrodia elata Blume [Orchidaceae]); Shengjiang (Zingiber officinale Roscoe [Zingiberaceae]) and Dazao (Ziziphus jujuba Mill. [Rhamnaceae]). Detailed ingredients are listed in Appendix A. These Chinese Herbal Medicine Slices were purchased from Kangmei Traditional Chinese Medicine Pieces Co., Ltd. (Guangdong, China) and identified by Professor Ping Ding (School of Pharmaceutical Sciences, Guangzhou University of Chinese Medicine, Guangdong, China). We deposited a voucher specimen (NO. 20201127002) in the public herbaria of Guangzhou University of Chinese Medicine. Herbal decoction of MLG was made according to conventional TCM decocting methods, and concentrating filtrates was extracted by condensing and stored at 4 °C.

### Animals

Kunming mice of specific-pathogen-free (SPF) grade (6–8 weeks old, weighing 20–25 g, male and female in equal number) were supplied and housed by the Experimental Animal Center of Guangzhou University of Chinese Medicine (No.SYXK (Yue) 2018–0001; No.SCXK (Yue) 2018–0034) and kept under a constant temperature (22–24°C), invariable humidity (50–60%) and a fixed 12 h light/dark cycle, with free access to food and water. Animal experiments followed the guidelines of the humane, ethical treatment of animals set forth by the World Health Organization and were approved by the Ethics Committee for Animal Studies of Guangzhou University of Chinese Medicine (NO. 20210316001).

### Reagents

The 56 percent liquor used in our study was provided by Baiyunbian Wine Industry Co. LTD. (Hubei, China); Colloidal Bismuth Pectin (CBP) was obtained from Guangdong Bidi Pharmaceutical Co. (Guangzhou, China); Hematoxylin eosin (H-E) staining reagents were purchased from Guangzhou Yiqiao Biotechnology Co., Ltd. (Guangzhou, China); kits used for determination of ADH, ALDH, T-SOD, MDA, NO, GSH-Pxt, MPO were purchased from Nanjing Jiancheng Biotechnology (Nanjing, China); ELISA kits for determining TNF-α (70-EK282/4–96) and IL-1β (70-EK201B/3–96) were purchased from MultiSciences (Lianke) Biotech Co. (Hangzhou, China); ELISA kits for mouse PGE2 (Prostaglandin E2) (E-EL-0034c) and mouse SS (Somatostatin) (E-EL-M1086c) were obtained from Elabscience biotechnology (Wuhan, China). BCA protein concentration determination kit and protein extraction kit were provided by Beijing Beyotime Biotechnology (Beijing, China). Trizol RNA isolation reagent, RevertAid Reverse Transcriptase and SYBR Green Real time PCR Master Mix was purchased from Thermo Fisher Scientific (NY, United States). The rabbit anti-Nrf2 antibody (AF0639), rabbit anti-HO-1 antibody (AF5393), rabbit anti-IκBα antibody (AF5002), rabbit anti-NF-κB p65 antibody (AF5006), rabbit anti-p-IκBα antibody (AF 2002), rabbit anti-p-p65 antibody (AF 2006), rabbit anti-SOD1 Antibody (AF5198), rabbit anti-SOD2/MnSOD Antibody (AF5144), rabbit anti-iNOS Antibody (AF0199), rabbit anti-nNOS Antibody (AF6249), rabbit anti-eNOS Antibody (AF0096), rabbit anti-Cox2 Antibody (AF7003), rabbit anti-p38 MAPK Antibody (AF6456) and rabbit anti-GAPDH antibody (AF7021) was obtained from Affinity Biosciences (OH, United States).

### Mice groupings and drug administration

Six mice per group were distributed among the 36 Kunming mice: model group, MLG-H (high-dose MLG-treated group, 20 g/kg body weight), MLG-M (medium-dose MLG-treated group, 10 g/kg body weight), MLG-L (low-dose MLG-treated group, 5 g/kg body weight), CBP (Colloidal Bismuth Pectin treatment group, 57 mg/kg body weight) and control group. Pretreatment of the mice with MLG and CBP was given by oral gavage after 24 h of fasting, while the other groups received the same dose of physiological saline in the same way. Two hours later, model group, MLG-L group, MLG-M group and MLG-H group were treated with alcohol (13.25 ml/kg body weight) by intragastric administration, the control group received the same volume of physiological saline. After 30 minutes, each group were administrated orally with corresponding drugs (MLG, CBP, or physiological saline) once again as described previously.

### Evaluation of the gastric ulcer index

Using isoflurane anesthesia, all mice were euthanized 5 hours after being given alcohol. Gastric tissues were collected and washed clean by 0.9% pre-cooling normal saline after gastric acidity (pH) was measured. Weight was measured for an empty stomach, and a stomach index was calculated. Also, the surface damage of gastric mucosa was observed, including bleeding, erosion and ulcer; the gastric ulcer index (GUI) was calculated macroscopically according to the Guth standard (Guth et al., 1979; El-Maraghy et al., 2015; Raish et al., 2018): we recorded one point for spot erosion; two points for erosion lengths up to 1mm; three points for erosion lengths between 1 and 2 mm, four points for those from 2 to 3 mm; five points for those more than 3 mm. If the erosion width is greater than 1 mm, the scores are doubled. Method for calculating ulcer inhibition rate: ulcer inhibition rate (%) = (GUI in control group - GUI in administration group)/GUI in control group × 100%.

### Gastric tissues histopathology assay

Fresh gastric tissues were fixed for 12–24 h at room temperature in 4% paraformaldehyde. Tissues were routinely dehydrated, transparent, and paraffin embedded before being sliced into sections of 4 or 5 μm thickness for haematoxylin and eosin staining. Hetopathologists reviewed slides under a 200X microscope under the supervision of an expert.

### Gastric tissues biochemical assays

A homogenate of 10% gastric tissues homogenates was obtained by homogenizing gastric tissues (100 mg) in 0.9% physiological saline, and the proteins in 10% homogenate were measured using the BCA protein assay kit (Beyotime Biotechnology, Beijing, China). The activity of T-SOD, GSH-Px, MDA, NO, MPO, ADH and ALDH were colorimetrically detected in gastric tissues homogenates using commercial kits (Nanjing Jiancheng Biotechnology, Nanjing, China) according to manufacturer directions.

### Enzyme-linked immunosorbent assay

TNF-α, IL-1β, PGE2 and SS levels in gastric tissues were detected using mouse ELISA kits. A 96-well plate was washed three times using PBST and subsequently incubated for 2 hours at room temperature with either 100 μl diluted serum or a standard. Add 100 μl of diluted detection antibody to each well and incubate for 1 h after washing the wells three times. The levels of TNF-α, IL-1β, PGE2 and SS levels in gastric tissues were read at 450 nm and the antibody concentrations were calculated based on a standard curve.

### Quantitative real-time polymerase chain reaction

Gastric tissues were homogenized in 1 ml of TRIzol and flash frozen in dry ice and stored at -80 °C until the total RNA of all samples were isolated according to the manufacturer’s protocol. Total RNA samples were reverse transcribed into complementary DNA (cDNA) with oligo dT18 primer using RevertAid Reverse Transcriptase and then were amplified using SYBR Real time PCR Master Mix (TAKARA, Shiga, Japan) in a Bio-rad fluorescence quantitative PCR instrument (Bio-rad, United States). The mRNA expression of Nrf-2, NF-κB, HO-1, TNFα, IL-1β, and COX2 were measured using reverse transcription quantitative real-time polymerase chain reaction (qRT-PCR) with GAPDH as internal reference gene. The reaction conditions were set as follows: one cycle at 50°C for 2 min and 95°C for 1 min; 40 cycles at 95°C for 15 s, 60°C for 15 s and 72°C for 30 s; with a dissolution curve being produced in the last cycle: 95°C, 15 s; 60°C, 1 min; 95°C, 15 s. RT-qPCR data were analyzed using relative quantification by standard curve method based on mRNA copy number ratio (R) of target gene versus reference genes GAPDH. The primers were designed with ABI Primer Express 3.0 software and synthesized by Suzhou Jinweizhi Biotechnology (Suzhou, China) and the sequences are shown in Table 1.

**TABLE 1:** Primer sequence of qRT-PCR.

Gene	Forward	Reverse
Nrf-2	5′-AGA​CAT​TCC​CAT​TTG​TAG​ATG​ACC-3′	5′-CTC​CAG​AGA​GCT​ATT​GAG​GGA​CT-3′
NF-κB	5′-CTG​GAA​GTC​ACA​TCT​GGT​TTG​AT-3′	5′-CAA​CCC​TCA​GCA​AAT​CCT​CTA​C-3′
HO-1	5′-ACC​GCC​TTC​CTG​CTC​AAC​ATT​G-3′	5′-CTC​TGA​CGA​AGT​GAC​GCC​ATC​TG-3′
TNFα	5′-TGT​CTC​AGC​CTC​TTC​TCA​TTC​C-3′	5′-GGT​CTG​GGC​CAT​AGA​ACT​GAT-3′
IL-1β	5′-TCC​ACC​TCA​ATG​GAC​AGA​ATA​TC-3′	5′-CCG​TCT​TTC​ATT​ACA​CAG​GAC​A-3′
COX2	5′-CGG​TGG​ATG​TGA​GTC​TAG​CTA​C-3′	5′-CGG​TGG​ATG​TGA​GTC​TAG​CTA​C-3′
GAPDH	5′-GAC​AAC​TCA​CTC​AAG​ATT​GTC​AGC-3′	5′-AGT​CTT​CTG​GGT​GGC​AGT​GAT-3′

Nrf-2, nuclear factor-erythroid 2-related factor 2; NF-κB, nuclear factor-kappa B; HO-1, heme oxygenase-1; TNFα, tumor necrosis factorα; IL-1β, interleukin-1β; COX2, cyclooxygenase two; GAPDH, glyceraldehyde-3-phosphate dehydrogenase.

### Western blot

Following the homogenization of the tissue samples, a BCA protein assay kit was used to determine the protein concentration. Protein was isolated from gastric mucosa isolated from mice stomach using a protein extraction kit (Beyotime Biotechnology, Beijing, China). Equal amounts of protein (30 mug) were separated by 10% SDS-polyacrylamide gel electrophoresis and were transferred onto a polyvinylidene fluoride (PVDF) membrane. Membranes were blocked in TBST (Tris-buffered saline, pH 7.6, 0.1% Tween 20) supplemented with 5% (w/v) BSA at room temperature (RT) for 1 h before incubation with rabbit anti-Nrf2 antibody (AF0639; 1:2000; Affinity, OH, United States), rabbit anti-HO-1 antibody (AF5393; 1:2000; Affinity), rabbit anti-IκBα antibody (AF5002; 1:2000; Affinity), rabbit anti-NF-κB p65 antibody (AF5006; 1:2000; Affinity), rabbit anti-p-IκBα antibody (AF 2002; 1:2000; Affinity), rabbit anti-p-p65 antibody (AF 2006; 1:2000; Affinity), rabbit anti-SOD1 Antibody (AF5198; 1:2000; Affinity), rabbit anti-SOD2/MnSOD Antibody (AF5144; 1:2000; Affinity), rabbit anti-iNOS Antibody (AF0199; 1:2000; Affinity), rabbit anti-nNOS Antibody (AF6249; 1:2000; Affinity), rabbit anti-eNOS Antibody (AF0096; 1:2000; Affinity), rabbit anti-Cox2 Antibody (AF7003; 1:2000; Affinity), rabbit anti-p38 MAPK Antibody (AF6456; 1:2000; Affinity) and rabbit anti-GAPDH antibody (AF7021; 1:2000; Affinity) overnight at 4°C, followed by incubation with a secondary antibody Goat Anti-Rabbit IgG (H + L) HRP (S0001; 1:10,000; Affinity) at RT for 1 h. The ECL enhanced chemiluminescence Plus Western blotting detection system was used for detecting immunoblots.

### Immunofluorescence analysis

Immunofluorescence staining was performed on gastric tissue sections for the detection of Nrf2 (rabbit anti-Nrf2, AF0639), HO-1 (rabbit anti-HO-1, AF5393), IκBα (rabbit anti-IκBα, AF5002), NF-κB (rabbit anti-NF-κB p65, AF5006) proteins according to standard protocols. Fresh gastric tissues were fixed in 4% paraformaldehyde for 24 h at 4°C, dehydrated in 15 and 30% sucrose solutions sequentially overnight at 4 °C. Frozen sections preserved in OCT were cut into 10 mum sections using frozen section machine (Leica, Weztlar, Germany). Slides were rehydrated in PBS for 10 min, blocked in 5% BSA/PBS/0.1% Triton-X 100 for 1 h, and then with primary antibodies overnight at 4°C. Slides were washed three times with PBS for 10 min each and incubated with Goat Anti-Rabbit IgG (H + L) FITC-conjugated (Affinity, S0008) for 1 h at room temperature and then mounted with DAPI counterstain (Vector Laboratories, Burlingame, CA, United States).

### Statistical analysis

Three independent experiments were conducted, and data from one representative experiment was shown. The data were presented as the mean ± standard deviation (SD) and were analyzed using one-way analysis of variance (ANOVA). Statistically significant difference was assumed to be *p* < 0.05, while *p* < 0.01 indicated a statistically significant difference of greater magnitude.

## Result

### Effects of MLG on gross evaluation in gastric mucosa

**FIGURE 1:**
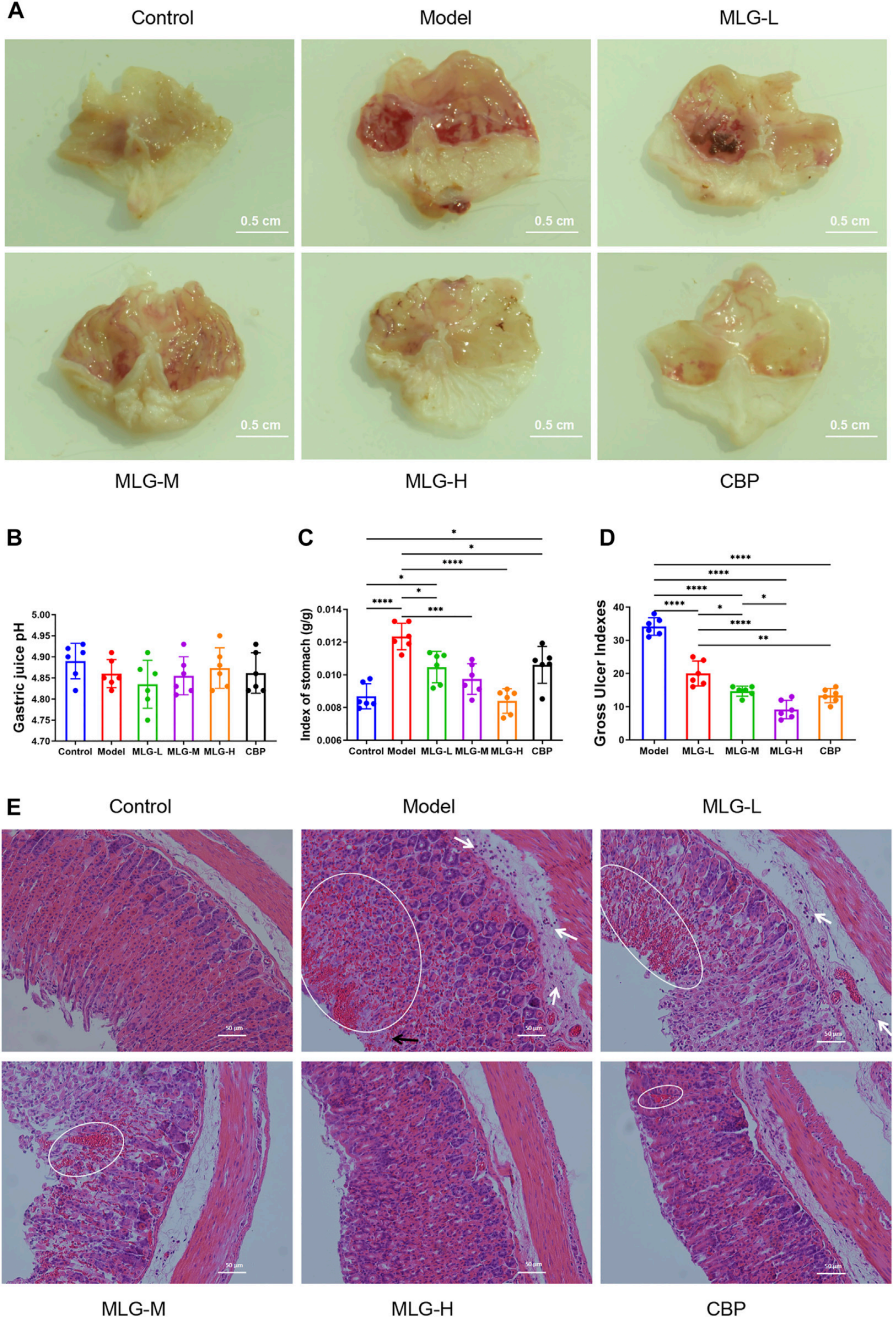
Gross evaluation in mice gastric tissue **(A)**; effects of MLG on gastric acidity **(B)**, stomach index **(C)** and gastric ulcer index **(D)**; H&E-stained in the gastric tissues of mice **(E)**, white arrows highlight inflammatory cell infiltration, black arrows highlight epithelial cell loss, and highlighted circles indicate hemorrhage. Control: negative control group; Model: the group induced by intragastric administration of ethanol (13.25 ml/kg BW); MLG-L: the group treated with low dose MLG (5 g/kg BW); MLG-M: the group treated with medium dose MLG (10 g/kg BW); MLG-H: the group treated with high dose MLG (20 g/kg BW); CBP: the group treated with CBP (57 mg/kg BW). Data was expressed as mean ± standard deviation (SD), n = 6 for each group. By one-way analysis of variance (ANOVA) test, *p < 0.05, ***p < 0.001, ****p < 0.0001.

As shown in Figure 1A, the gastric mucosal surfaces of mice in the normal group were pink, smooth and glossy, while the gastric mucosae of mice in model group were observed with extensive bleeding, edema, accompanied by local ulcer and erosion. Compared with model group, mice treated with MLG and CBP showed varied degrees of gastric tissue injury, most obvious in MLG-L group, but mild in the other three. The gastric mucosa of mice treated with high-dose MLG and CBP were light pink, with basically smooth surface and no bleeding and erosion, which showed obviously relieved in the injury of gastric mucosa.

### Effects of MLG on gastric acidity, stomach index and gastric ulcer index

According to Figures 1B–D, there was no significant difference between the groups when it came to gastric acidity (pH) values (*p* > 0.05). From the comparison with the control group, strikingly higher stomach indexes and ulcer gastric indexes are associated with acute alcohol exposure, however, the indexes in the groups treated with MLG and CBP were significantly lower than those of the model group (*p* < 0.05), especially in the MLG-M group (*p* < 0.001) and the MLG-H group (*p* < 0.0001). The ulcer inhibition rate was mushroomed markedly in MLG groups and CBP group compared to the model group, exhibiting an obvious dose-effect relationship in MLG’s gastroprotective effects.

### Effects of MLG on mice gastric tissues histopathology

As is shown in Figure 1E, the gastric tissue structure in control group was normal with a continuous and integral mucosa, and the cells were arranged neatly with clear morphology. Gastric tissues in the Model group displayed obvious alterations primarily caused by epithelial cell loss, structural disorders of glandular tissues, submucosal edema, hemorrhage, and infiltration of inflammatory cells. Nevertheless, the pathological changes in MLG treated groups were milder to a dose-dependent extent. Moreover, both the MLG-M and MLG-H groups maintain gastroprotective effects comparable to that of the CBP group. Our results showed that MLG possessed a significant gastric protection effect and could effectively alleviate the pathological changes of gastric tissues in acute ethanol exposure mice.

### Effects of MLG on the metabolism of alcohol in the gastric tissues

**FIGURE 2:**
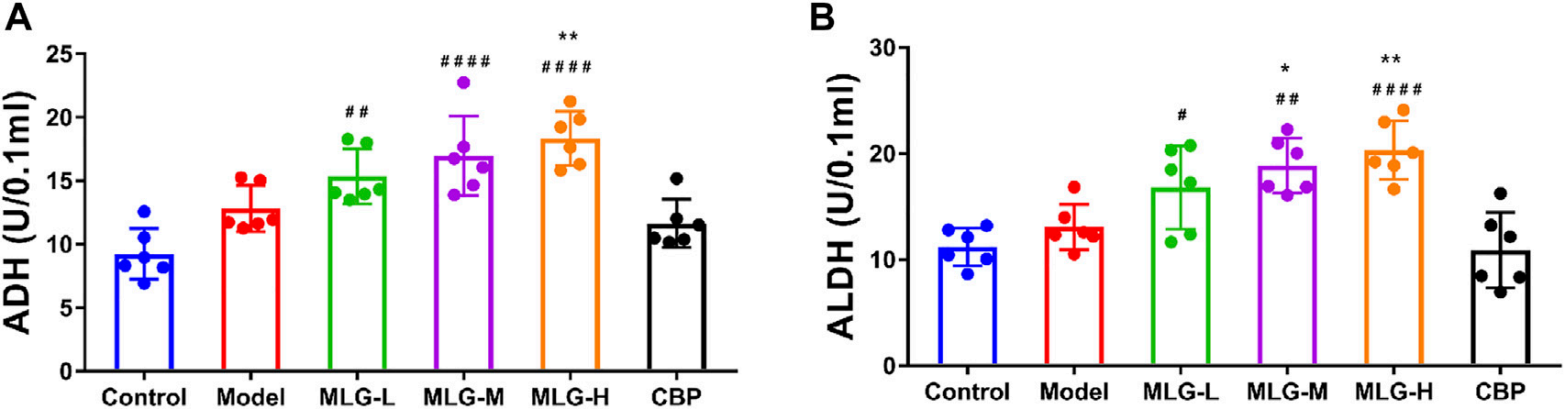
ADH activity **(A)** and ALDH activity**(B)** in mice gastric tissues. Control: the group administered zero ethanol; Model: the group administered ethanol intragastrically (13.25 ml/kg BW); MLG-L: low-dose (5 g/kg body weight) MLG-treated group; MLG-M: medium-dose (10 g/kg body weight) MLG-treated group; MLG-H: high-dose (20 g/kg body weight) MLG-treated group; CBP: the group receiving Colloid Bismuth Pectin (57 mg/kg body weight). Each group’s data was expressed as mean x standard deviation (SD), n = 6. By one-way analysis of variance (ANOVA) test, ^*^ *p* < 0.05, ^**^ *p* < 0.01 vs. model group; ^#^ *p* < 0.05, ^##^ *p* < 0.01, ^####^ *p* < 0.0001 versus the control group.

The activities of ADH and ALDH in gastric tissue were slightly elevated in response to acute alcohol exposure (Figure 2) due to the short-term and superfluous alcohol intake, but there was no significant statistical difference (*p* > 0.05). Comparatively to the model group, ADH and ALDH levels in gastric tissue of the MLG treated groups can be seen a remarkable upward trend, especially in MLG-M (*p* < 0.05) and MLG-H (*p* < 0.01) groups with no significant changes showed in CBP group (*p* > 0.05). Our results demonstrated MLG was extraordinarily effective on accelerating the metabolism of alcohol.

### Effects of MLG on T-SOD, MDA, GSH-Px, NO and MPO levels in gastric tissues

**FIGURE 3:**
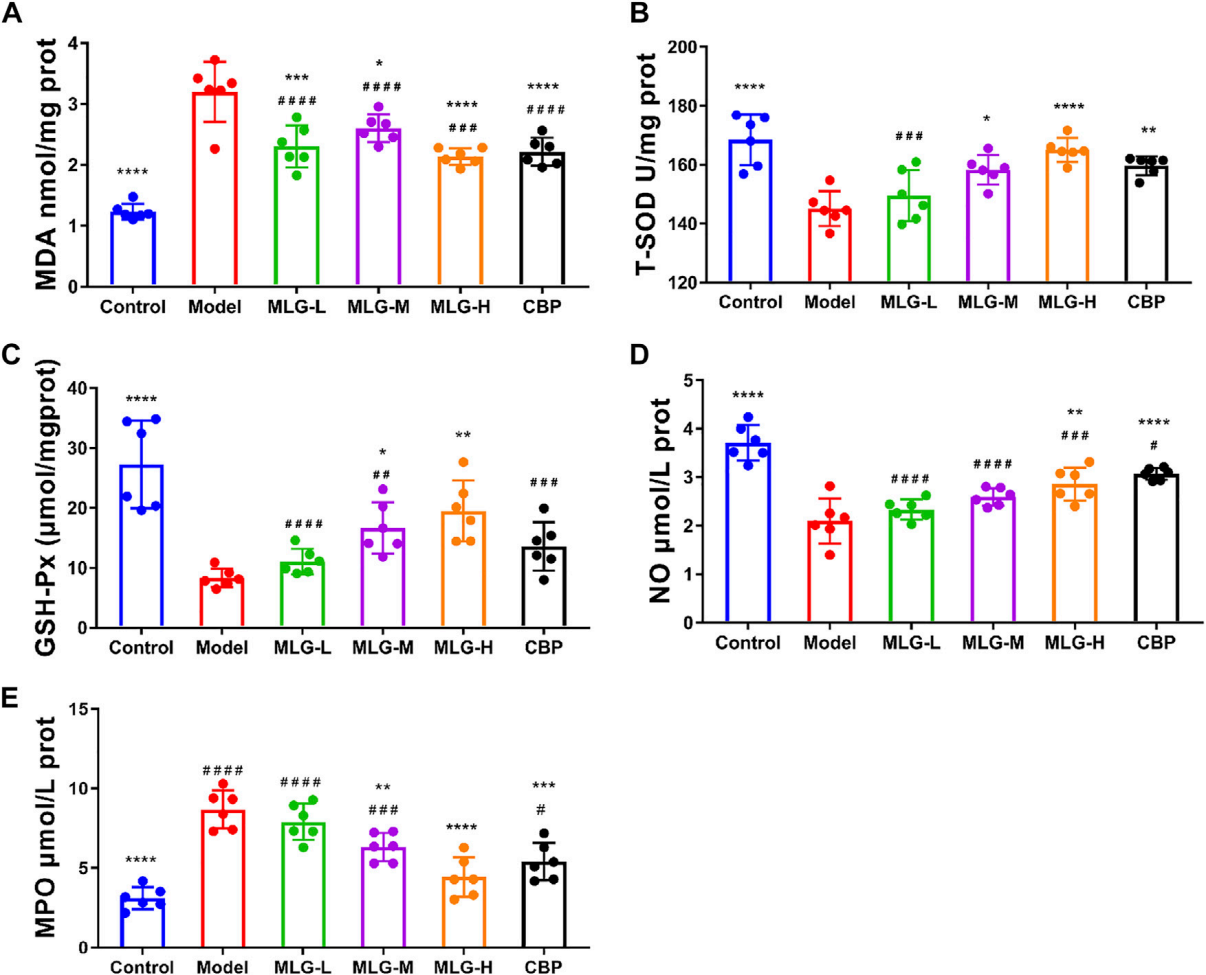
MDA **(A)**, T-SOD **(B)**, GSH-Px **(C)** NO **(D)** and MPO**(E)** levels in mice gastric tissues. Control: negative control group; Model: the group induced by intragastric administration of ethanol (13.25 ml/kg BW); MLG-L: the group treated with low dose MLG (5 g/kg BW); MLG-M: the group treated with medium dose MLG (10 g/kg BW); MLG-H: the group treated with high dose MLG (20 g/kg BW); CBP: the group treated with CBP (57 mg/kg BW). Each group’s data was expressed as mean ± standard deviation (SD), n = 6. By one-way analysis of variance (ANOVA) test, *p < 0.05, **p < 0.01, ***p < 0.001, ****p < 0.0001 versus the model group, ^#^ *p* < 0.05, ^##^ *p* < 0.01, ^###^ *p* < 0.001, ^####^ *p* < 0.0001 versus the control group.

As can be seen in Figure 3, acute alcohol administered in mice significantly down-regulated T-SOD, GSH-Px, and NO levels (*p* < 0.0001) but up-regulated MDA and MPO level (*p* < 0.0001). Whereas the mice treated with MLG and CBP, especially in CBP (*p* < 0.01 or *p* < 0.0001), MLG-M (*p* < 0.05) and MLG-H (*p* < 0.01 or *p* < 0.0001) groups, had a reversed trend in comparison to the Model group. In the MLG-H group, particularly, the effect proved to be comparable or even more efficient than that of the CBP group, with T-SOD, GSH-Px and NO levels shooting up to 165.03 ± 4.08, 19.51 ± 5.06 and 2.86 ± 0.34, respectively, while MDA and MPO level dropping to 2.14 ± 0.14 and 4.43 ± 1.24 respectively. Our results revealed that MLG could abate the oxidative damage of gastric tissues caused by acute alcoholism via cutting the production of oxidative damage products and improving the levels of antioxidant enzymes.

### Effect of MLG on mRNA expression of Nrf2, NF-κB, HO-1, TNF-α, IL-1β, and COX2 in gastric tissues

**FIGURE 4:**
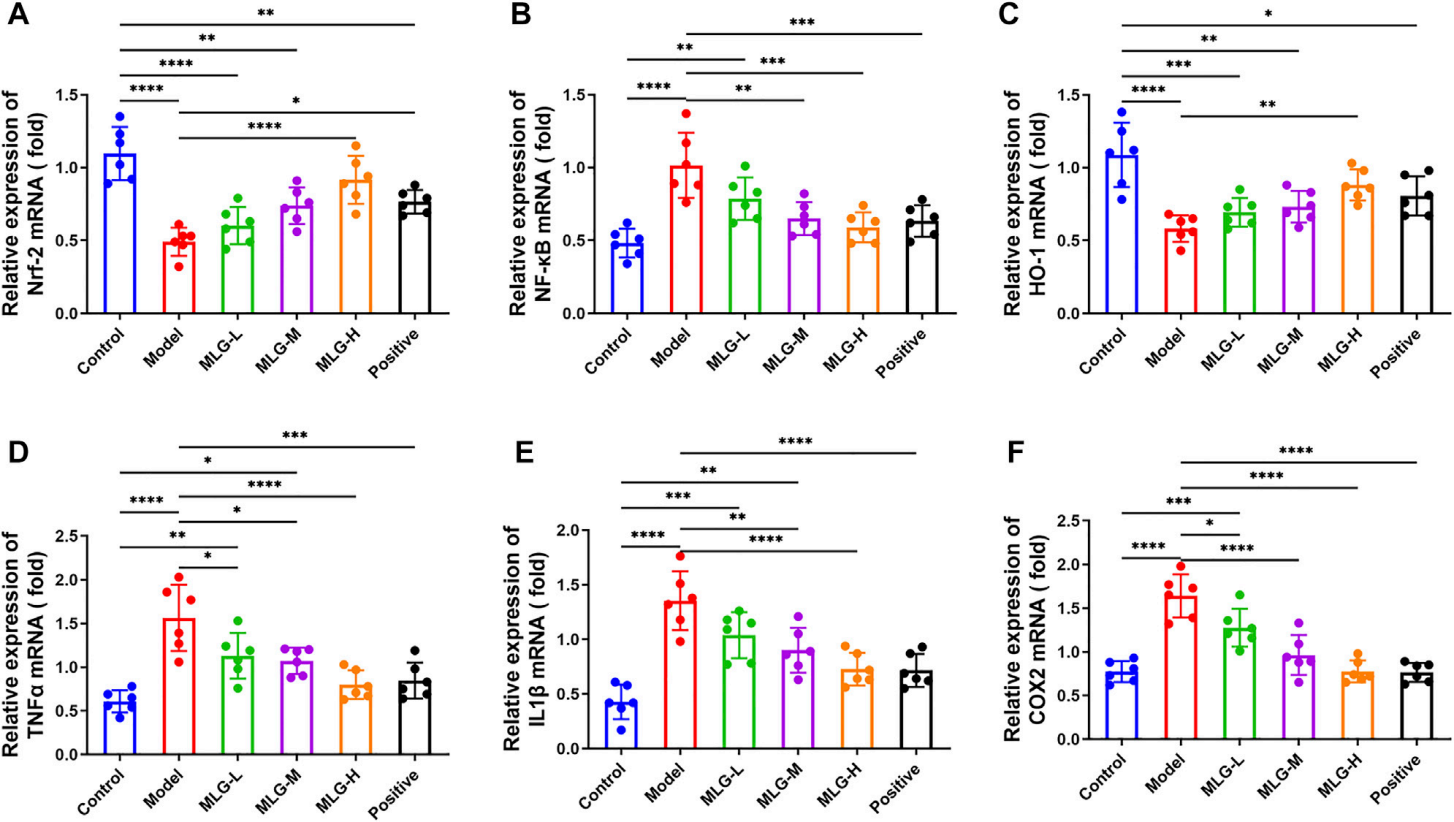
Effect of MLG on mRNA levels of Nrf-2 **(A)**, NF-κB **(B)**, HO-1 **(C)**, TNFα **(D)**, IL-1β **(E)**, COX2 **(F)** in mice gastric tissues. Control: negative control group; Model: the group induced by intragastric administration of ethanol (13.25 ml/kg BW); MLG-L: the group treated with low dose MLG (5 g/kg BW); MLG-M: the group treated with medium dose MLG (10 g/kg BW); MLG-H: the group treated with high dose MLG (20 g/kg BW); CBP: the group treated with CBP (57 mg/kg BW). Each group’s data was expressed as mean ± standard deviation (SD), n = 6. By one-way analysis of variance (ANOVA) test, *p < 0.05, **p < 0.01, ***p < 0.001, ****p < 0.0001 vs. each group.

As shown in Figure 4, quantitative polymerase chain reaction analysis indicated that acute alcohol administered in mice induced significant enhance (*p* < 0.0001) in the mRNA expression levels of NF-κB, TNF-α, IL-1β and COX2, with the Nrf2 and HO-1 mRNA expression levels, especially in MLG-M and MLG-H groups, showing a downward trend (*p* < 0.0001). The mRNA expression levels of NF-κB, TNF-α, IL-1β and COX2 in the gastric tissue of the MLG-treated mice, for the MLG-H group particularly, dropped to 0.59 ± 0.1, 0.80 ± 0.16, 0.72 ± 0.15 and 0.78 ± 0.13, respectively, were lower than those in the model group (*p* < 0.001 or *p* < 0.0001) and comparable to or even lower than that of the positive group. Meanwhile, the mRNA expression levels of Nrf2 and HO-1 mRNA in MLG-H group raise to 0.92 ± 0.16 and 0.88 ± 0.11, were higher than those in the model group (*p* < 0.01 or *p* < 0.0001).

### Effects of MLG on TNF-α, IL-1β, PGE2 and SS levels in gastric tissues

**FIGURE 5:**
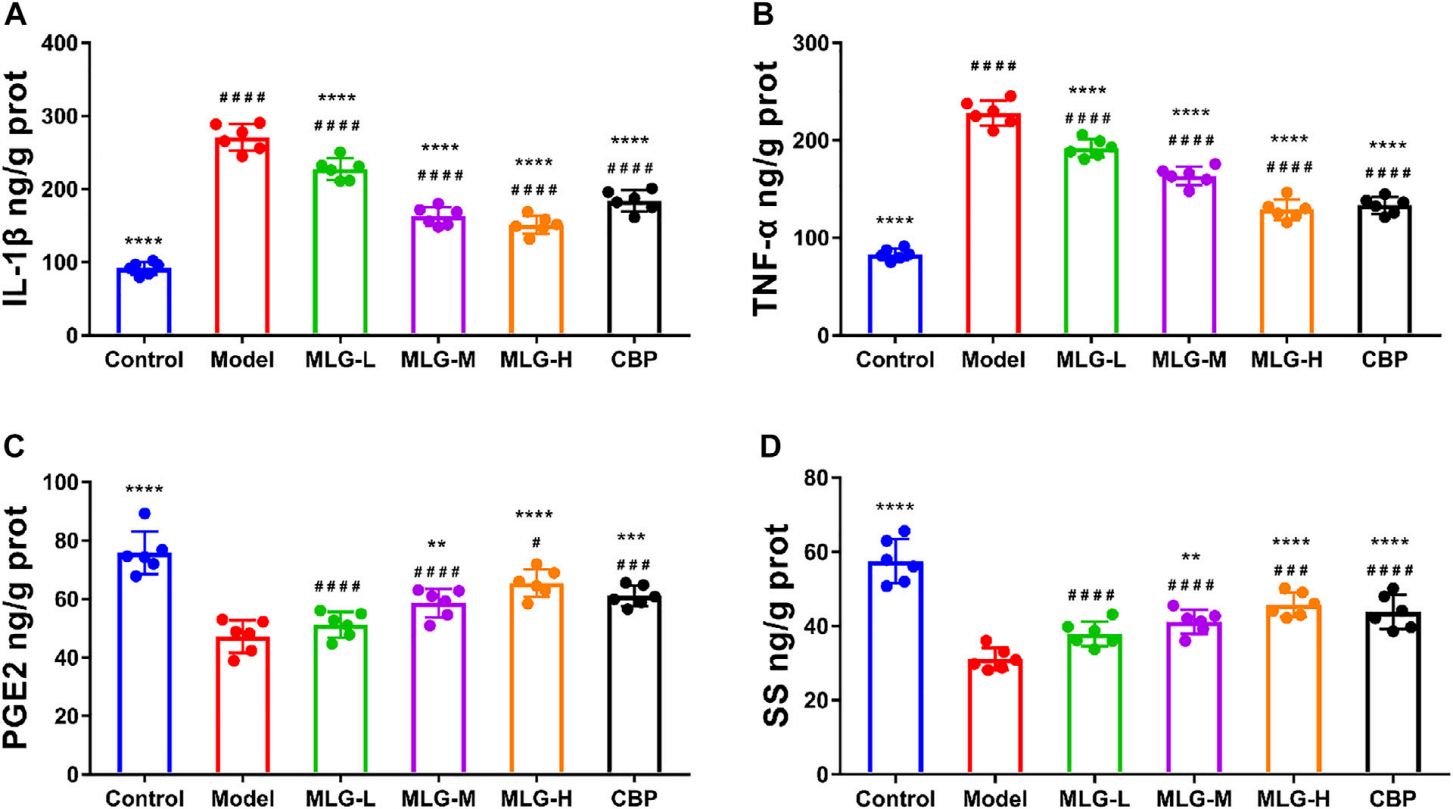
IL-1β **(A)**, TNF-α **(B)**, PGE2 **(C)** and SS **(D)** levels in mice gastric tissues. Control: negative control group; Model: the group induced by intragastric administration of ethanol (13.25 ml/kg BW); MLG-L: the group treated with low dose MLG (5 g/kg BW); MLG-M: the group treated with medium dose MLG (10 g/kg BW); MLG-H: the group treated with high dose MLG (20 g/kg BW); CBP: the group treated with CBP (57 mg/kg BW). Each group’s data was expressed as mean ± standard deviation (SD), n = 6. By one-way analysis of variance (ANOVA) test, **p < 0.01, ***p < 0.001, ****p < 0.0001 versus the model group, ^#^ *p* < 0.05, ^###^ *p* < 0.001, ^####^ *p* < 0.0001 versus the control group.

As determined by ELISA assays, acute alcohol exposure significantly increased TNF-α and IL-1β protein levels (*p* < 0.0001) and reduced the protein levels of PGE2 and SS (*p* < 0.0001) synchronously (Figure 5). The levels of TNF-α and IL-1β were dwindled in MLG treated groups (*p* < 0.0001), but the levels of PGE2 and SS were remarkably increased (*p* < 0.01 or *p* < 0.0001) comparatively to the model group, which indicated that MLG could effectively relieve the expression of TNF-α and IL-1β to inhibit the inflammatory response of gastric tissues caused by acute ethanol exposure, protect gastric mucosa and increase blood supply of gastric tissues. Evidently, acute alcohol exposure can stimulate the secretion of inflammatory mediators, such as TNF-α and IL-1β, and inhibit the flow of the gastric mucosal microcirculation. In contrast, when MLG was administered, this trend was reversed.

### Effect of MLG on Nrf-2/HO-1/NF-κB signaling pathway in gastric tissues

**FIGURE 6:**
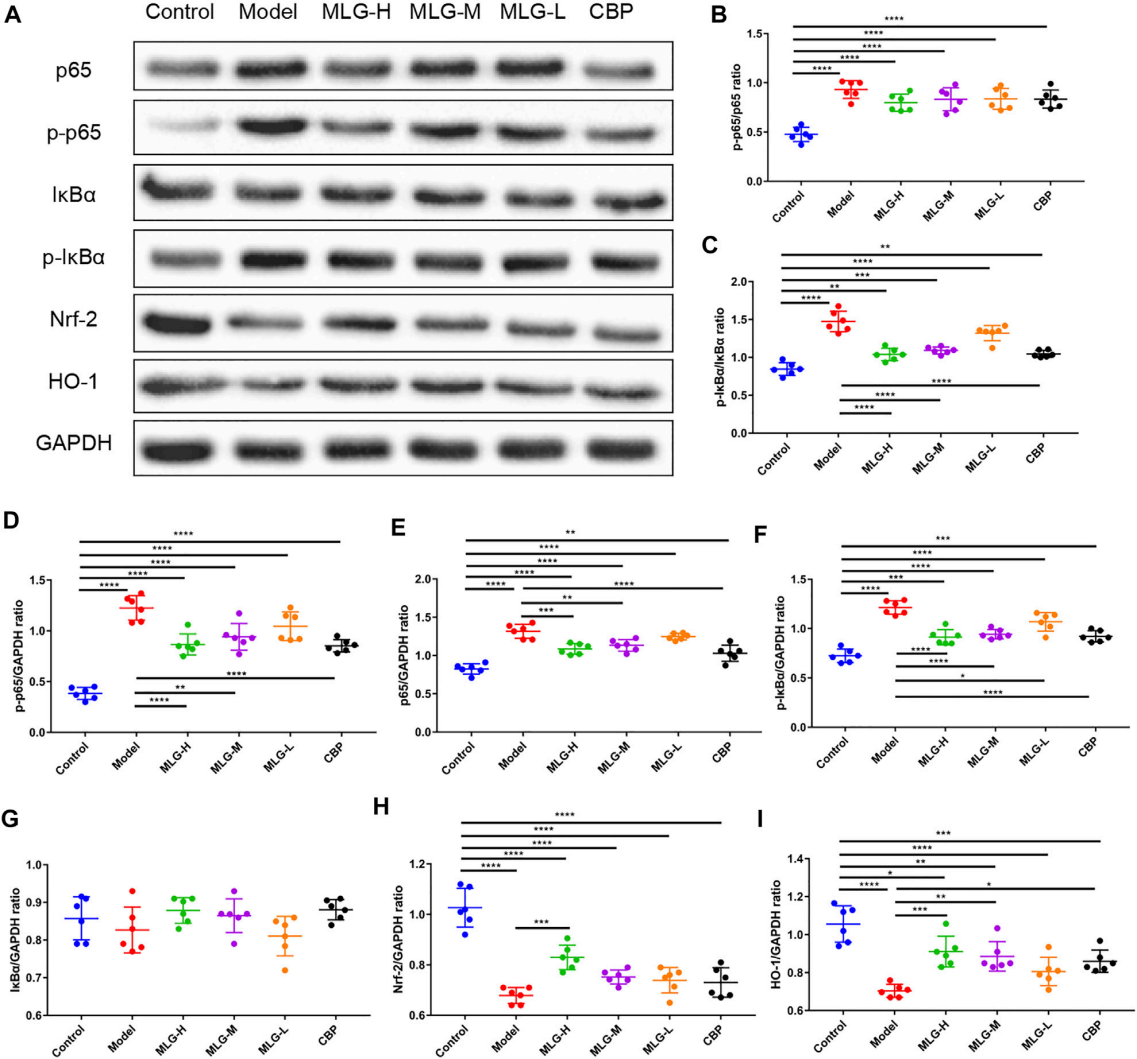
Effect of MLG on NF-κB/Nrf2/HO-1 signaling pathway in ethanol-induced gastric lesions mice. **(A)** Some representative western blot bands. Protein levels of p-p65/p65 **(B)**, p-IκBα/IκBα **(C)**, p-p65/GAPDH **(D)**, p65/GAPDH **(E)**, p-IκBα/GAPDH **(F)**, IκBα/GAPDH **(G)**, Nrf2/GAPDH **(H)**, HO-1/GAPDH **(I)** in mice gastric tissues. Control: the group administered zero ethanol; Model: the group administered ethanol intragastrically (13.25 ml/kg BW); MLG-L: low-dose (5 g/kg body weight) MLG-treated group; MLG-M: medium-dose (10 g/kg body weight) MLG-treated group; MLG-H: high-dose (20 g/kg body weight) MLG-treated group; CBP: the group receiving Colloid Bismuth Pectin (57 mg/kg body weight). Each group’s data was expressed as mean ± standard deviation (SD), n = 6. By one-way analysis of variance (ANOVA) test, *p < 0.05, **p < 0.01, ***p < 0.001, ****p < 0.0001 vs. each group. Whole page of western blot can be found in Supplementary Figures S4–S6, S9, S11–S13, respectively.

**FIGURE 7:**
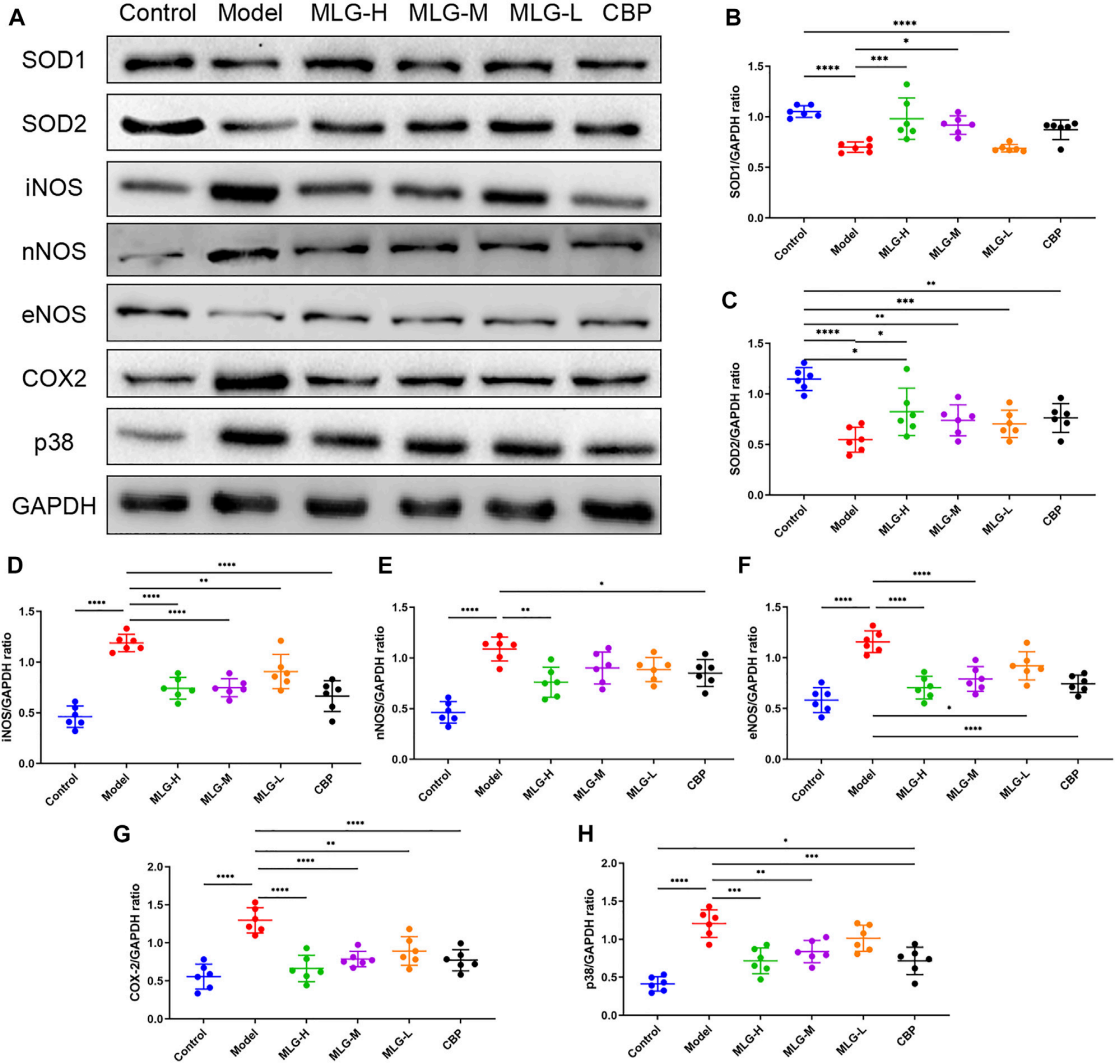
Effect of MLG on some protein levels in ethanol-induced gastric lesions mice. Some representative western blot bands **(A)**. Protein levels of SOD1/GAPDH **(B)**, SOD2/GAPDH **(C)**, iNOS/GAPDH **(D)**, nNOS/GAPDH **(E)**, eNOS/GAPDH **(F)**, COX2/GAPDH **(G)**, p38/GAPDH **(H)** in mice gastric tissues. SOD1, Superoxide dismutase one or Cu/Zn-superoxide dismutase; SOD2/Mn SOD, superoxide dismutase 2. Control: the group administered zero ethanol; Model: the group administered ethanol intragastrically (13.25 ml/kg BW); MLG-L: low-dose (5 g/kg body weight) MLG-treated group; MLG-M: medium-dose (10 g/kg body weight) MLG-treated group; MLG-H: high-dose (20 g/kg body weight) MLG-treated group; CBP: the group receiving Colloid Bismuth Pectin (57 mg/kg body weight). Each group’s data was expressed as mean ± standard deviation (SD), n = 6. By one-way analysis of variance (ANOVA) test, *p < 0.05, **p < 0.01, ***p < 0.001, ****p < 0.0001 vs. each group. Whole page of western blot can be found in Supplementary Figures S1, S3, S7, S8, S10, S14, S15, respectively.

As illustrated in Figure 6 and Figure 7, the levels of Nrf-2, HO-1, SOD1 (Cu/Zn-SOD), SOD2 (SOD2/Mn SOD) and eNOS proteins were reduced after alcohol exposure (*p* < 0.0001) in the mice gastric tissues, whereas the levels of p65, p-p65, p-p65/p65 (the ratio is 0.93 ± 0.09), p-IκBα, p-IκBα/IκBα (the ratio is 1.47 ± 0.14), iNOS, nNOS, COX2 and p38 were elevated (*p* < 0.0001). In MLG treated groups, however, the inclination was observed to be reversed. Our results suggested that Nrf-2, HO-1, SOD1 (Cu/Zn-SOD), SOD2 (SOD2/Mn SOD) and eNOS expression were significantly increased (*p* < 0.001, *p* < 0.01 or *p* < 0.05) in mice gastric tissues by the treatment of MLG after alcohol exposure, whereas p65, p-p65, p-IκBα, p-p65/p65, p-IκBα/IκBα, iNOS, nNOS, COX2, and p38 levels decreased partially but significantly (*p* < 0.0001, *p* < 0.001, *p* < 0.01 or *p* < 0.05). We also found that this regulation of Nrf-2/HO-1/NF-κB signaling pathway with the treatment of MLG was in a dose-dependent manner. Together, the results revealed that MLG has potent anti-oxidative and anti-inflammatory properties via the suppression of the NF-κB phosphorylation and activation of Nrf-2/HO-1 antioxidant pathway to attenuate the oxidative damage and inflammatory response and improve the defensive factors in response to ethanol-induced gastric lesions.

### Effects of MLG on mice gastric tissues immunofluorescence staining

**FIGURE 8:**
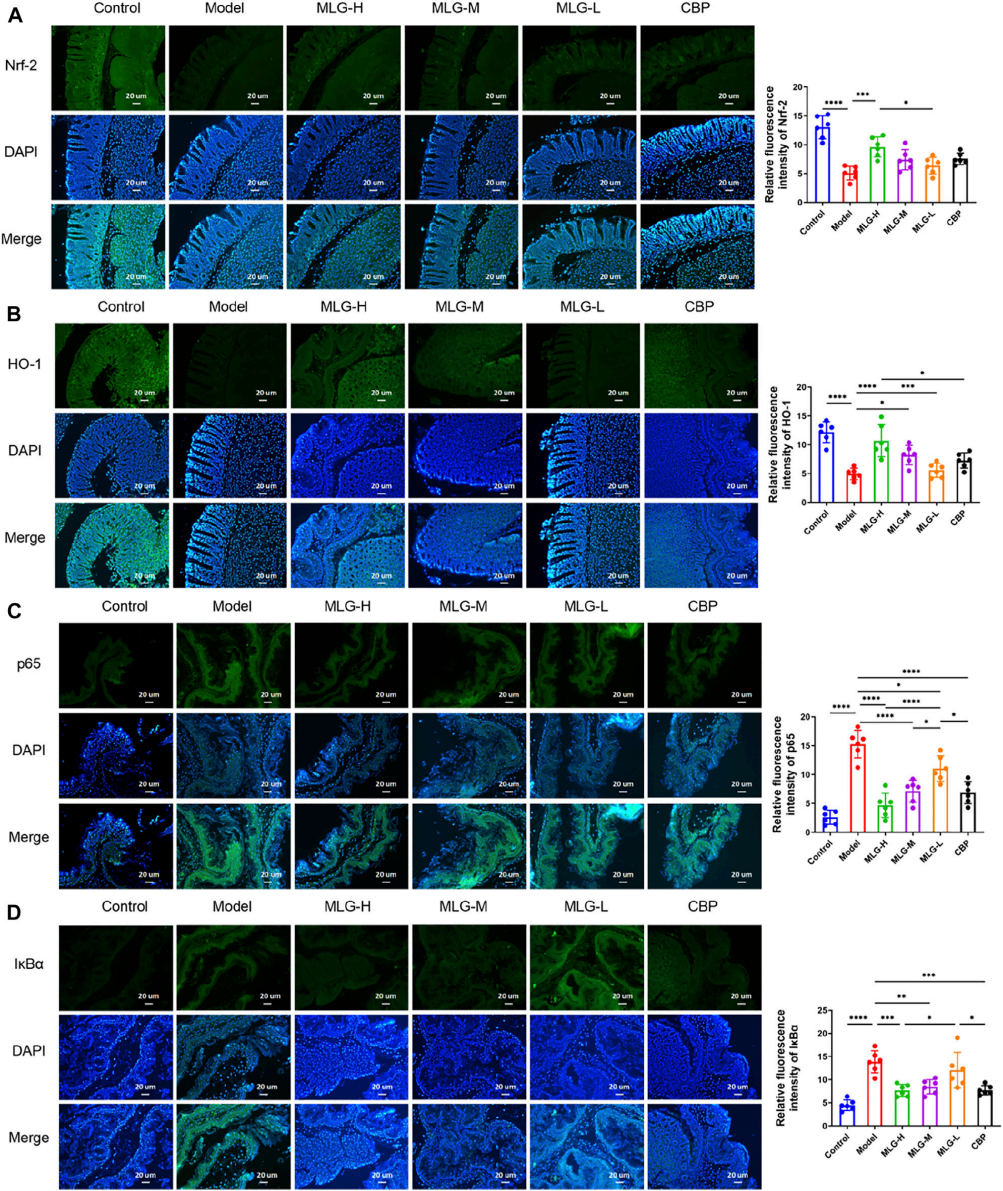
Representative immunofluorescence pictures and relative quantitative analysis on mice gastric tissues of Nrf-2 **(A)**, HO-1 **(B)**, p65 **(C)** and IκBα **(D)** The blue immunfluorescence is DAPI, showing nuclei. Graphs showing relative quantitative analysis of NF-κB/Nrf2/HO-1 signaling pathway-related proteins, performed with ImageJ software. Control: the group administered zero ethanol; Model: the group administered ethanol intragastrically (13.25 ml/kg BW); MLG-L: low-dose (5 g/kg body weight) MLG-treated group; MLG-M: medium-dose (10 g/kg body weight) MLG-treated group; MLG-H: high-dose (20 g/kg body weight) MLG-treated group; CBP: the group receiving Colloid Bismuth Pectin (57 mg/kg body weight). Each group’s data was expressed as mean ± standard deviation (SD), n = 6. By one-way analysis of variance (ANOVA) test, *p < 0.05, ***p < 0.001, ****p < 0.0001 vs. each group.

Immunofluorescence staining was performed on gastric tissue sections for the detection of Nrf2, HO-1, IκBα and NF-κB proteins (as shown in Figure 8). The immunofluorescence results showed the expression of IκBα and NF-κB was increased while the expression of Nrf2 and HO-1 was decreased in model group. The relative quantitative analysis of NF-κB/Nrf2/HO-1 signaling pathway-related proteins displayed a reversal trend after MLG treatment. The expression of Nrf2 and HO-1 were up-regulated (*p* < 0.0001, *p* < 0.001 or *p* < 0.05) and simultaneously, IκBα and NF-κB expression were down-regulated (*p* < 0.0001, *p* < 0.001, *p* < 0.01 or *p* < 0.05), showing the results that agree with the qPCR and western blot. Moreover, the relative fluorescence intensity of Nrf2 and HO-1, the key components of the cellular antioxidant defense system, in MLG-H group were increased considerably (*p* < 0.001, *p* < 0.0001). Noticeably, the treatment of MLG could activate Nrf2/HO-1 signaling pathway and inhibit NF-κB signaling pathway, reversing the trend of acute alcohol exposure.

## Discussion

Ethanol-induced gastric lesion is a ubiquitous emergency worldwide, and the request for more effective therapeutic drugs still keeps impending. Although MLG shows great competence in treating alcohol-induced symptoms and gastric Lesions in clinical practice, the exact mechanism remains ambiguous.

In our study, we mainly discussed that: 1) MLG could prevent acute alcohol intoxication and promote wakefulness after acute ethanol exposure; 2) MLG showed a significant gastric protection effect, which is able to drastically abate the gastric ulcer index, raise the ulcer inhibition rate and alleviate the pathological changes of gastric tissues in mice effectively; 3) ADH and ALDH levels in gastric tissue of the MLG treated groups showed a upward remarkably, which revealed a significant effect of MLG on accelerating the alcohol metabolism; 4) MLG could reduce the oxidative damage for gastric tissues by decreasing the production of oxidative damage products and increasing the levels of antioxidant enzymes; 5) MLG might aid the repair of the damaged gastric mucosa by inhibiting the release of inflammatory mediators and accelerating microcirculation of the gastric mucosa, NO and PGE2 possibly involved in the process; 6) MLG could attenuate the oxidative damage and inflammatory response and improve the defensive factors, which might be associated with the activation of the Nrf2/HO-1 signaling and the repression of the NF-κB signaling pathway. In general, MLG could significantly accelerate the metabolism of alcohol and attenuate ethanol-induced gastric mucosal lesions in mice, which might be put down to the activation of alcohol metabolizing enzymes, the inhibition of oxidative damage and the maintenance of gastric mucosa involving the regulation of Nrf-2/HO-1/NF-κB signaling pathway. It is worth mentioning that the protection effects of MLG for ethanol-induced gastric lesions appears to be comparable to the classical gastric mucosal protection agent Colloidal Bismuth Pectin (CBP). Moreover, MLG could significantly accelerate the metabolism of alcohol and reduce the toxicity of alcohol, a property not shared by CBP.

ADH and ALDH are crucial enzymes involved in alcohol metabolism, which convert about 90% of ethanol to acetaldehyde, and the resulting acetaldehyde is further oxidized into harmless acetic acid. This biological process plays a critical role in the rate of alcohol metabolism and the detoxification of alcohol (Gizer et al., 2011; Meng et al., 2019). We recorded the elevation of the activities of ADH and ALDH in gastric tissues after alcohol administration in mice, which could be considered as an adaptive response to alcohol stimulation. In comparison, the raise of ADH and ALDH levels in gastric tissues were more significant in mice treated with MLG, and a dose-dependent manner is showed with the increment of MLG-M and MLG-H groups being especially arresting. The marvelous results revealed that MLG can stimulate alcohol metabolism, which is convincingly supported by the enhancive ADH and ALDH levels in the gastric tissues of MLG-treated group.

Ethanol, a recognized necrotizing agent, could directly irritate and damage the gastric mucosa, making the gastric mucosal epithelium more susceptible to degeneration and necrosis. We observed that, compared with the model group, MLG-treated groups exhibited lower gastric ulcer index, higher ulcer inhibition rate and milder gastric tissue injury, suggesting that MLG could efficaciously ameliorate ethanol-induced gastric lesions in mice. The histopathological examination of gastric tissues shown a similar tendency. A series of pathological changes that indicated severe gastric lesions, including epithelial cell loss, hemorrhage, structural disorders of glandular tissues, submucosal edema, and inflammatory cells infiltration, could be seen in the model group. The administration of MLG certainly mitigated the situation in a dose-dependent manner. The pathological changes including epithelial cell loss, structural disorder of glandular tissues, submucosal edema and hemorrhage were observed to a milder extent within all MLG-treated group, and, strikingly, the effect of MLG-H group is comparable (and even better) to CBP.

The pathogenesis for ethanol-induced gastric lesions is manifold and complicated, but chief among them is oxidative stress-mediated aggravated inflammatory response. It is well established that ethanol and its metabolite acetaldehyde attack the gastric mucosa causing microcirculatory disturbance and hypoxia, along with the propagation of the inflammatory cascade due to the depletion of mucus and bicarbonate, eventually resulting in cellular necrosis and the excessive production of ROS (Périco et al., 2020). It is ROS that is responsible for the pathogenesis of ethanol-induced gastric lesions mediated by oxidative stress (Sistani Karampour et al., 2019), which interferes with the antioxidant systems of endogenous cells in mucosa, induces leukocyte recruitment and boosts inflammatory response. In the gastric mucosa, the relation, between ROS generation and antioxidant protection mediated through antioxidant enzymes, maintains homeostasis. Oxidative stress triggers a cascade, bringing on the excessive production of ROS and the accumulation of malondialdehyde (MDA). MDA is a significant marker of ROS peroxidation due to its role as a primary lipid peroxidation end product, which manifests the damage of ROS on gastric mucosal directly and is mediated by the development of gastric lesions (Yuksel et al., 2012). ROS also stimulates inhibitor Kappa B (IκB) kinase to trigger the proteasomal breakdown of IκBα, the release of NF-κB p65, and, at length, the activation of NF-κB. NF-κB serves as a catalyst for the transcription of a host of inflammatory cytokines and chemokines, being responsible for the high expression of inflammatory factors including TNF-α, IL-6, and IL-1β (Wardyn et al., 2015; Mitchell et al., 2016; Akanda and Park, 2017). TNF-α and IL-1β, multifunctional pro-inflammatory cytokines, conduce to the activation and migration of inflammatory cells into the gastric mucosa as well as the gastric inflammatory process (Sugimoto et al., 2009). The secretion of inflammatory mediators, including TNF-α and IL-1β, is consistent with the activation of polymorphonuclear neutrophil leukocytes, inflammatory infiltration of lymphocytes, and macrophages in gastric tissue after acute ethanol exposure (Bagheri et al., 2015).

When oxidative stress transpires, antioxidant defense system acts actively as well to scavenge ROS, defend against oxidative stress in cell and guard the body. Nuclear factor erythroid 2-related factor 2 (Nrf2) is a crucial transcriptional regulator for cellular defenses against oxidant-associated damage. Excessive ROS phosphorylates Nrf2 and dissociates it from its inhibitor, Kelch-like epichlorohydrin-associated protein 1 (Keap1); the activated Nrf2 will translocate to the nucleus and bind with Maf proteins, to stimulate antioxidant response elements (AREs) and activate downstream antioxidant enzymes such as heme oxygenase-1 (HO-1), superoxide dismutase (SOD), catalase (CAT) and glutathione peroxidase (GSH-Px) (Takei et al., 2015; Kim et al., 2022). HO-1, a stress-inducible enzyme, is considered as a reliable anti-oxidative and cytoprotective agent, which catalyzes the breakdown of heme into equimolar amounts of biliverdin, ferrous iron and carbon monoxide (Puentes-Pardo et al., 2020). Signaling pathways associated with inflammation and oxidative stress, mainly including Nrf2 and NF-κB, control the expression of HO-1. SODs are universal and essential enzymes for organisms that live in the presence of oxygen, and they are widely recognized as the first hurdle in the fight against oxygen free radicals (Wang et al., 2018). Characterized by requiring for different catalytic metal ions, SODs are divided into three classes in various organisms including copper/zinc SOD (Cu/Zn SODs), manganese/iron SOD (Mn SOD/Fe SODs), and nickel SOD (Ni SODs) (Wang et al., 2018). GSH-Px is highly concentrated in gastric tissue, which is also a vital member of antioxidant system, and gastric mucosa GSH loss may further exacerbate lipid peroxidation along with cell membrane and gastric mucosa damage (Zhang et al., 2013).

The mitogen-activated protein kinases (MAPKs) are important cellular signaling molecules transferring various extracellular signals to intracellular responses by sequential phosphorylation cascades. Several distinct but parallel subgroups have been identified, including C-Jun N-terminal kinase (JNK), Extracellular signal-regulating kinases (ERKs) and p38 MAPK(Bak et al., 2016). MAPKs are involved in a variety of cell biological processes, such as cell proliferation, differentiation, stress response, apoptosis, cell migration, and survival. p38 MAPK is closely related to multiple pathways related to oxidative stress, which is activated and phosphorylated under environmental influence. It is well founded that p38 MAPK is activated and phosphorylated by oxidative stress. Tissue damage and other external stimuli will trigger the secretion of multiple pro-inflammatory cytokines such as TNF-α, IL-1β, as well as IL-6, and subsequently, the activation of p38(Chaparro Huerta et al., 2008). The relation between p38 MAPK and NF-κB has been reported that a variety of pro-inflammatory factors can give rise to the modified IκBs degradation and the p65 translocation into the nucleus directly or via the activated p38-MAPK to activate NF-κB pathway (Xie et al., 2019). Recent studies have found that high expression of P38 can threaten the integrity of gastrointestinal mucosa, and, in contrast, the inhibition of p38 MAPK phosphorylation can regulate claudin expression, thereby improving the function of gastrointestinal mucosal epithelial barrier (Carrozzino et al., 2009). Increased TNF-α level leads to the disruption of tight epithelial cells and the phosphorylation of P38. HO-1 can keep the barrier intact by blocking tight junction disruption caused by TNF-α and phosphorylation of ERK, P38, and JNK(Zhang Z et al., 2021). Furthermore, MAPK cascades are found to be involved in HO-1 activation. It has been reported that p38 MAPK takes part in the protein synthesis of HO-1 as well as the activation and translocation of Nrf2 to help the body resist oxidative stress (Bak et al., 2016).

The destruction of gastric mucosal integrity can be considered as the disequilibrium between multiple endogenous aggressive factors (such as hydrochloric acid, leukotrienes, and ROS) and defensive factors, including a functional mucus-bicarbonate barrier, nitric oxide (NO), prostaglandins (PG), mucosal microcirculation, antioxidants and some growth factors (Ibrahim et al., 2016). Prostaglandin E2 (PGE2) and NO are postulated to be important defenders of the gastric epithelial mucosal barrier. PGE2, a production of arachidonic acid, is well recognized as a protective factor essential for the repair of damaged gastric mucosa. PGE2 synthesis involves two steps: arachidonic acid is converted into prostaglandin H2 (PGH2) by cyclooxygenase-1 (COX-1) and cyclooxygenase-2 (COX-2), and then PGH2 is isomerized to PGE2 by prostaglandin E synthases (Kloska et al., 2020). As a recognized vasodilator factor, PGE2 has been shown to inhibit platelet aggregation and thrombosis, as well as increase blood flow to the mucosa surface of the stomach, restrain gastric acid secretion, regenerate cells, mediate adaptive immune protective functions and promote mucosal repair (Chen et al., 2016; Fang et al., 2019). COX isoenzymes mainly exist in two isoforms: the constitutive form (COX-1) and the inducible form (COX-2). COX-2 is restricted at the site of inflammation, acting as a proinflammatory enzyme, and its expression could vary drastically as reacting to inflammatory stimuli or growth factors (Zhang J et al., 2021). It is well known that NO plays an indispensable role in maintaining the integrity of the gastric mucosal, which can repair damaged gastric mucosa by regulating gastric mucosal blood flow, acid and alkaline secretion as well as mucus secretion (Ismail Suhaimy et al., 2017). Paradoxically, NO also triggers mucosal damage. NOS, playing a pivotal role in the synthesis of NO by transforming l-arginine to l-citrulline, mainly can be divided into three forms including neuronal NOS (nNOS, type I), inducible NOS (iNOS, type II), and endothelial NOS (eNOS, type III), whereby each of them participates in multiple biological processes. nNOS and eNOS can both be grouped into constitutive NOS (cNOS), characterized by calcium dependence, while iNOS is calcium independent (Kumar and Chanana, 2017; Mohamed et al., 2022). In the digestive system, cNOS-generated NO and iNOS-produced NO are related to contradictory outcomes, with the former one displaying the cytoprotective effect and the latter being cytotoxic (Motawi et al., 2007). eNOS-generated NO facilitates the healing of ulcer via scavenging the damaging free radicals, eliciting angiogenesis, increasing vasodilation, and attenuating leukocyte infiltration, thereby aiding mucous secretion and epithelial tissue integrity restoration (Khattab et al., 2001; Abd El-Rady et al., 2021). iNOS usually generates NO in response to a wide range of stimuli, such as the production of inflammatory cytokines (IL-1β, TNF-α). iNOS-produced NO is found to be highly involved in gastric damage, which reacts with superoxide directly to form a potent cytotoxic oxidant, peroxynitrite (Abdel-Raheem, 2010; Kumar and Chanana, 2017). Somatostatin (SS), an important gastrointestinal hormone, could directly inhibit the secretion of acid and indirectly suppress the secretion of histamine and gastrin to protect the gastric mucosa (El-Salhy et al., 2014). Myeloperoxidase (MPO) is mainly found in neutrophils and therefore becomes the specific marker of neutrophils. During the initial inflammatory response, the accumulation of neutrophils results in high expression of MPO, and the activity of MPO can in turn reflect the severity of inflammation in tissues (Al-Quraishy et al., 2017). Moreover, MPO can induce oxidative stress by stimulating the production of reactive oxygen species (ROS) and active nitrogen (RNS) (Chen et al., 2020).

As our observation in the current study, ethanol exposure induced a drastic augment in the MDA level, while GSH-Px and T-SOD concentrations suffered a dramatic decline, signifying the enhancement of oxidative stress as well as lipid peroxidation. This, in turn, serves as a catalyst in the wreck of the antioxidant defense system, making the gastric mucosa more susceptible to injury. MLG has significant antioxidant activity, represented by significantly inhibiting the elevation of MDA while promoting the T-SOD and GSH-Px levels even in a small dose (Figures 5A–C), which effectively reduced the production of oxidative damage products, inhibited the activity of antioxidant enzymes, and protected the mucosa of the stomach from alcohol-induced damage. Our results demonstrated that MLG could inhibit the secretion of inflammatory mediators (TNF-α, IL-1β, iNOS, MPO, p38-MAPK, and COX-2) and up-regulate the activities of defensive factors (PEG2, NO, eNOS, and SS) in gastric tissues after ethanol exposure, which could attenuate the inflammation response and accelerate the flow of gastric mucosal microcirculation to repair the damaged gastric mucosa. On this account, we speculated that MLG could reduce the infiltration of inflammatory lymphocyte cells in gastric mucosa, which is consistently supported by the amelioration of inflammatory cells infiltration in mice gastric tissue histomorphology examination of MLG-treated groups. We found that MLG treatment markedly blocked the NF-κB expression and suppressed the phosphorylation of p65 and IκBα indicating that MLG alleviates ethanol-induced gastric lesions by inhibition of the ROS-mediated inflammatory signaling cascade. Synchronously, Nrf2 and HO-1 levels were observed up-regulated in MLG treatment groups, which demonstrated that the cytoprotective factors were activated to defense the oxidative stress damage and improve the gastric healing of ethanol-induced gastric lesions at the same time. In other words, MLG might have defensive effects on gastric mucosa by counterbalancing ROS generation and antioxidant defense systems in the gastric mucosa, which might rely on reducing the oxidative stress and strengthening the antioxidant defense concurrently via the molecular cross-talk of NF-κB/Nrf-2/HO-1. Taken together, our results demonstrated that MLG, as natural antioxidants, could help protect the gastric mucosa from oxidative stress and improve its defenses in order to moderate gastric mucosal microcirculation and preserve gastric mucosa.

## Conclusion

Taken together, our study delivered a point of view that MLG could effectively protect the gastric mucosa of mice against the gastric damage of ethanol-induced gastric lesions, mainly presented as the activation of alcohol metabolizing enzymes, attenuation of oxidative damage and inflammatory response, up-regulation of the defensive factors and improvement of vasodilation. Most glaring of all, MLG could remarkably accelerate the metabolism of alcohol and reduce the toxicity of alcohol, a property not shared by CBP. The gastroprotective effect of MLG on ethanol-induced gastric lesions may be achieved through the weakened of damage factors (TNF-α, IL-1β, iNOS, MPO, p38-MAPK, and COX-2) and the enhancement of defensive factors (PEG2, NO, eNOS, and SS) in gastric tissues involving NF-κB/Nrf2/HO-1 signaling pathway. We further confirmed that MLG has a strong potential in preventing and treating ethanol-induced gastric lesions.

## Data Availability

The original contributions presented in the study are included in the article/Supplementary Material, further inquiries can be directed to the corresponding author.

## Abbreviations

ADH, alcohol dehydrogenase; ALDH, aldehyde dehydrogenase; CBP, Colloidal Bismuth Pectin; Elisa, Enzyme-linked immunosorbent assay; GSH-Px, glutathione peroxidase; GUI, gastric ulcer index; H&E, hematoxylin and eosin; IL-1β, interleukin-1β; MDA, malondialdehyde; MLG, Modified Lv-dou-Gan-cao decoction; MLG-L, low dose of MLG; MLG-M, medium dose of MLG; MLG-H, high dose of MLG; NO, nitric oxide; TCM, traditional Chinese medicine; PGE2, prostaglandin E2; ROS, reactive oxygen species; SS, somatostatin; T-SOD, total superoxide dismutase; TNFα, tumor necrosis factor-α.

## Appendix A: Contents of MLG.

**Table:**

Chinese name	Accepted name	Weight (g)	Medicinal part	Plantchemical composition
Lvdou	Vigna radiata (L.) R.Wilczek [Fabaceae]	50	Seeds	sitosterol, beta-carotene, vitamin-e, vitexin_qt, vitexin_qt PQN
Gancao		10	Roots and rhizomes	Glycyrol, Glycyrol, licopyranocoumarin, shinpterocarpin, Phaseol, Licochalcone B, glyasperin F, Inermine, Vestitol, Glyasperins M
	Glycyrrhiza glabra L. [Fabaceae]			
Dangshen		10	Root	Perlolyrine, Perlolyrine, glycitein, glycitein
	Codonopsis pilosula (Franch.) Nannf. [Campanulaceae]			
Shanyao		15	Tuber	diosgenin, diosgenin, Isofucosterol, Stigmasterol, hancinone C, Denudatin B, Kadsurenone, hancinol, (-)-taxifolin
	Dioscorea oppositifolia L. [Dioscoreaceae]			
Chuanxiong	Conioselinum anthriscoides 'Chuanxiong' [Apiaceae]	10	Rhizome	sitosterol, Myricanone, Mandenol, wallichilide, senkyunone, Perlolyrine FA
Gansong		15	Roots and rhizomes	(2R)-5,7-dihydroxy-2-(4-hydroxyphenyl)chroman-4-one, acaciin, acacetin, sitosterol, sitosterol
	Nardostachys jatamansi (D.Don) DC. [Caprifoliaceae]			
Dingxiang		10	Flower Bud	Strictosamide_qt, ZINC03860434, beta-sitosterol, kaempferol, Stigmasterol, quercetin
	Syzygium aromaticum (L.) Merr. & L.M.Perry [Myrtaceae]			
Jiangbanxia		10	Tuber	Baicalin, (3S,6S)-3-(benzyl)-6-(4-hydroxybenzyl)piperazine-2,5-quinone, beta-d-Ribofuranoside, xanthine-9, Stigmasterol, Stigmasterol, 10,13-eicosadienoic, Cycloartenol, beta-sitosterol, 24-Ethylcholest-4-en-3-one, Cavidine, baicalein, coniferin, gondoic acid
	Pinellia ternata (Thunb.) Makino [Araceae]			
Baishao		30	Root	11alpha,12alpha-epoxy-3beta-23-dihydroxy-30-norolean-20-en-28,12beta-olide, paeoniflorgenone, (3S,5R,8R,9R,10S,14S)-3,17-dihydroxy-4,4,8,10,14-pentamethyl-2,3,5,6,7,9-hexahydro-1H-cyclopenta[a]phenanthrene-15,16-dione, Lactiflorin, paeoniflorin, paeoniflorin_qt, albiflorin_qt, benzoyl paeoniflorin, Mairin, beta-sitosterol, sitosterol, kaempferol, (+)-catechin
	Paeonia lactiflora Pall. [Paeoniaceae]			
Lianfang		10	Receptacle	procyanidin, Hyperin, quercetin
	Nelumbo nucifera Gaertn. [Nelumbonaceae]			
Tianma		10	Tuber	gastrodin, P-hydroxybenzyl alcohol, Parisin E, Parisin A, Parisin B, Parisin C
	Gastrodia elata Blume [Orchidaceae]			
huang Jiezi	Brassica juncea (L.) Czern. [Brassicaceae]	10	Seeds	Uniflex BYO, 2-(2-phenylethyl)-6-[[(5S,6R,7R,8S)-5,6,7-trihydroxy-4-keto-2-(2-phenylethyl)-5,6,7,8-tetrahydrochromen-8-yl]oxy]chromone, Sinoacutine
Shengjiang		5	Fresh rhizome	beta-sitosterol, 6-methylgingediacetate2, Stigmasterol, Stigmasterol, Dihydrocapsaicin
	Zingiber officinale Roscoe [Zingiberaceae]			
Dazao		5	Pulp and seeds	Spiradine A, Mauritine D, Moupinamide, Ziziphin_qt, Fumarine, malkangunin, Mairin, (+)-catechin, (-)-catechin, Daechuine S6, Daechuine S6, Daechuine S7, Stigmasterol, (S)-Coclaurine
	Ziziphus jujuba Mill. [Rhamnaceae]			
